# Supplementary material for: TacticAI: an AI assistant for football tactics
Source: Nat Commun. 2024 Mar 19;15:1906. doi: 10.1038/s41467-024-45965-x (PMC10951310; doi:10.1038/s41467-024-45965-x)
Supplement: Supplementary file 1 — Supplementary Information [file 41467_2024_45965_MOESM1_ESM.pdf]

# Supplementary Information for "TacticAI: an AI assistant for football tactics"

Zhe Wang[1+*], Petar Veličković[1+*], Daniel Hennes[1+], Nenad Tomašev[1], Laurel Prince[1], Michael Kaisers[1], Yoram Bachrach[1], Romuald Elie[1], Li Kevin Wenliang[1], Federico Piccinini[1], William Spearman[2], Ian Graham[3], Jerome Connor[1], Yi Yang[1], Adrià Recasens[1], Mina Khan[1], Nathalie Beauguerlange[1], Pablo Sprechmann[1], Pol Moreno[1], Nicolas Heess[1], Michael Bowling[4], Demis Hassabis[1] and Karl Tuyls[5*]

[1] Google DeepMind, 6-8 Handyside Street, London N1C 4UZ, UK
[2] Liverpool FC, AXA Training Centre, Simonswood Lane, Kirkby, Liverpool L33 5XB, UK
[3] Work completed while at Liverpool FC
[4] University of Alberta, 3-58 Athabasca Hall, 9119 - 116 St NW, Edmonton T6G 2E8, Canada
[5] Work completed while at Google DeepMind
[+]Contributed equally to this work
[*]Corresponding authors: zhewang@google.com, petarv@google.com, ktuyls@gmail.com

## Supplementary Discussion

In the following, we summarise the closest state of the art that pursues the same objective of football corner kick tactics or pass prediction, or which more broadly devises team-sport strategy modelling techniques that could be transferred to the setting considered in this work. To the best of our knowledge, there are no previous graph neural network models proposed specifically for corner kick tactics. Sports analytics research that addresses player transfers and match statistics is out of scope, as we here focus on methods for in-game tactics.

Earlier work has generated a significant body of research on identifying key properties of a game given diverse sources of data, and on using these to provide tactical analysis and suggestions [1, 2, 3, 4, 5, 6]. One recent example is a system for determining whether a match is in-play or interrupted, called "in-game status", by applying random forests and AdaBoost to process time-continuous spatio-temporal data such as player positions [7], with similar methods having been proposed for American football [8].

Other methods leverage game data to characterize players behaviour, such as their passing style [9]. Such information can later feed downstream applications that may take as input a representation of a player's historical behaviour. Similarly, pass prediction systems may use game data to predict the probability of a pass in a given game state [10].

A more closely related research line analyzes soccer videos to determine shot event probabilities, which may serve as a proxy for player performance, by examining spatio-temporal relations and prediction uncertainty [11]. The method processes videos to obtain the players' team and position

information, followed by a graph convolutional recurrent neural network to capture the latent features, and a Bayesian neural network approach for predicting the final shot probabilities. There are several differences between this prior work and our proposed approach. The utilised frame preprocessing step in [11] that extracts the player positioning for subsequent analysis comes with a set of limitations. First, the approach is only able to detect field players, not goalkeepers. This is because players in the frame are classified into the respective teams based on the shirt colour information, and goalkeepers tend to wear different colours, introducing the risk of being mistaken for referees. Substitutions are disambiguated based on the pitch coordinates. Second, for the same reason, the method in [11] doesn't actually resolve player identity, only team affiliation for the detected players on the pitch. Without the identity information, it is not possible to cross-reference meta-data on player height, weight, or other potentially relevant features that may be otherwise available for analysis. As our ablation experiments have shown (see Table 4 and 5), this data plays a relevant role in our predictive model, and the use of such features can improve the model's predictive performance. The model proposed in [11] tries to work around this limitation by incorporating visual features directly, which may provide a noisy approximation to some (but not all) such metadata. In contrast, our proposed approach, which has full information on field players and goalkeepers alike, can be flexibly extended by domain experts to incorporate an arbitrary set of additional features of relevance, in subsequent model development and refinement.

Complementary to our use case, traditional statistical machine learning methods (logistic regression, XGBoost, and Random Forests) have been applied to derived player tracking features to evaluate salient features of defensive tactics that are most likely to result in winning the ball from the attacking side [12]. Through the application of these methods the authors conclude that direct pressure on the player leading the ball, coupled with compact defensive positioning and numerical superiority in the local area around the ball are most likely to result in a successful outcome for the defensive side. While insightful, the overall performance of these statistical predictors limits the potential utility of this method for iteratively improving individual tactics in a robust way. In contrast, our proposed approach offers the set piece coaches the ability to apply a more fine-grained level of analysis for tactic refinement on a case-by-case basis, rather than focus of high-level aggregate insights. Defensive play was also investigated in [13], where the authors proposed Tactical Graph Networks and showed that they were capable of reaching comparable performance compared to more complex deep learning methods. This work also emphasizes the importance of representation learning, which is a theme that we explore in our study as well.

Accurately identifying the salient patterns of corner kick tactics from player trajectories is the key prerequisite for downstream tasks in football analytics, including evaluating the effectiveness of the tactics, improving their performance and designing counter-tactics. Through leveraging deep learning, previous research demonstrated that the outcomes of in-game events, including shots and passes [14, 15, 16], are predictable using the spatio-temporal tracking data of player trajectories. The graph neural network model proposed in [14] was shown to be able to identify moments of interest in the football game when the likely receiver of the potential pass from the ball carrier changes, due to a change in circumstance on the pitch. However, the model was not as good at predicting shots conditioned on the pass being made to an attacking player, with the GNN having the same performance as the baseline MLP model considered in the paper, rendering pass completion the primary focus of the work. As for models that take the raw visual features as input, their end-to-end nature comes with a cost, given that it may prove challenging for such models to learn reliable feature extractors within these end-to-end pipelines on the volume of data that is usually available, and to be robust across all the variations in the conditions of play. While the

utility of these approaches may increase with the increasing amount of available recordings, at the moment it remains useful to detach the two components in the analysis, and ensure a higher quality of the input features that go into the predictive network.

There are also other examples of learning techniques for modelling team sports. The Generative Relational Inference Network (GRIN) [17] follows the VAE framework to learn disentangled representations for each agent in a dynamic scene, rather than a fixed frame as studied here. It encodes each node into a latent representation, comprising an intra-agent intention and an inter-agent relation, through a graph convolution network (GCN). The inter-agent relations under go message passing through graph attention layers, the result of which is combined with intra-agent intention to form the reconstruction loss. Rich latent edge features are inferred from the networks. Although there is no group-invariant layers or data augmentation, experiments show that this method could discover disentangled factors from basketball players, and interventions on these factors produced interpretable variations in the predicted player trajectories.

Using NBA games spacio-temporal tracking data, DeepHoops [18] presents a Deep Learning architecture that estimates the impact of all micro-actions in on the outcome of Basketball plays. In particular, it allows to evaluate the contribution of individual off-ball events to the success of a possession. Graph attention networks with temporal convolutional layers have recently been proposed as a way of accurately predicting the NBA player in-game performance [19]. Graph neural networks have also recently been used within the FAUPA-ML framework for improving the understanding of factors behind plays in handball [20], by successfully utilising the existing expert knowledge and scaling it to large datasets.

In [21] a tree-search method (Kernel Regression UCT) has been applied to discover curling strategies in self-play. The authors show that counterfactual justifications of actions taken by the model can be used to teach humans non-trivial concepts of curling as demonstrated in a user-study.

# Supplementary Methods

## Case Study Design

As previously described in the Results section, we designed an in-depth case study to evaluate the benefits of TacticAI's predictive and generative capabilities. We designed four specific tasks which we offered to five experts affiliated with Liverpool FC. To avoid biasing our raters, we have kept all details of these tasks strictly hidden from them prior to conducting the case study. Further, for all corner situations shown we did not reveal the team or player identities—only the players' positions and velocities at the moment the corner kick was taken, and whether they are on the attacking or defensive team. We remark that we do not reveal the heights and weights of the players to the raters. This was done deliberately to avoid over-cluttering the provided inputs, and we have performed ablations (in Supplementary Tables 4–5) which demonstrate that excluding these features does not critically limit TacticAI's predictive power.

**Task 1: Does TacticAI generate *realistic* tactical adjustments?** To answer this question, we started from a dataset of 50 corners from the Premier League, which have been held-out from TacticAI's training set (Supplementary Figure 3). For a subset of those corners, we have applied TacticAI's generative head to propose alternate tactics for either minimising or maximising shot probability, and used TacticAI's suggestion in place of the original corner. We then asked our raters to judge whether each of these corners was real or generated. Through discussions with the

raters after the task was done, and observing their comments, we noted a variety of strategies were employed to detect realism:

- Most raters chose to decide realism based on detecting whether there was something markedly unusual in the setup, for example a strategy that left opposing players in clear space.
- An alternate strategy, employed by one rater, was to decide realism based on whether the situation could have plausibly happened, even if it had a clearly suboptimal tactical setup. This approach ended up tagging most of the examples as real.
- Raters who specialise in corner kick analysis preferred a "retrieval" based strategy, wherein they would rate a corner as real if they were able to recall from memory that exact setup.

In spite of this variety of approaches, and as discussed in the Results section, all of the raters were generally confused as to which samples were real, and they unanimously highlighted how challenging they found this task.

**Task 2: Does TacticAI predict *plausible* receivers?** To answer this question, we reused the same 50 corners leveraged for Task 1, and simultaneously asked our raters which of the attacking team's players were most likely to make first contact with the ball (Supplementary Figure 3). The raters were allowed to provide as little or as many players as they wanted to—and we cross-referenced their proposals with TacticAI's top three suggested receivers. Generally, we found significant variability in the amount of players proposed by different raters—some opted to suggest 3–5 receivers on almost all situations studied, whereas others did not want to suggest more than one receiver on most occasions. As discussed in the Results section, in spite of this diversity of approach, the top-3 predictions made by TacticAI largely agreed with the rater estimates, and there was no significant difference between TacticAI's accuracy over real and generated setups, indicating that TacticAI could be robustly used to predictively analyse both real and simulated corner kicks. And, as highlighted by several of the raters, such a system would prove useful to a football analyst: a receiver predictor can be used to indicate which players may need to be especially targeted defensively, as well as to evaluate whether corners in already-played games have unfolded in a manner which is expected.

**Task 3: Can TacticAI be used to retrieve usefully *similar* corners?** To answer this question, we leveraged 50 reference corners from the Premier League, held-out from TacticAI's training set (Supplementary Figure 4). For each of these corners, we retrieved one other Premier League corner that was closest to it—either in TacticAI's graph-level embedding space, or in terms of the distance of the raw player features in the input space. This decision allowed us to not only measure whether TacticAI's embeddings can be used to mine for similar corners, but also to assess whether they provide an edge over a simpler mining heuristic which is purely position based. We asked our raters whether they find the retrieved corner to be usefully similar to the reference corner; that is, whether showing these two corners side by side would be judged as useful. The raters once again assumed a diverse set of approaches for deciding which features of the two corners were salient enough to be useful. Some of the common considerations mentioned by the raters included: whether the corner is in- or out-swinging, existence of a short corner option, zonal vs. man-marking approach, counting the number of players in the 18-yard box, positioning of the goalkeeper, and assessing whether the two corners likely had the same attacking or defending team, or had come

from the same game. Similarly as for the previous tasks—from the discussion in the Results section we can conclude that, despite the wide variety of salient features considered, the raters generally found TacticAI's retrievals to be superior to the positional heuristic. They also highlighted the high utility of such a retrieval system: it can be used by analysts and coaches to discover and prepare for other teams' common routines, as well as to discover new ideas and variations on a particular corner that they might otherwise have overlooked. It has also been stressed that the "top-1" retrieval study employed here is likely too strict. While it was necessary to restrict attention to only one retrieved corner here in order to keep the raters' workload manageable, in practice, an analyst may wish to analyse up to 10–20 retrieved corners for every reference corner. Given that TacticAI already has a favourable score in the top-1 regime, we anticipate its utility to only compound if deployed for real-world use.

**Task 4: Does TacticAI generate *useful* tactical adjustments?** To answer this question, we once again gathered a dataset of 50 held-out corners from the Premier League (Supplementary Figure 5). For each of them, we have used TacticAI's guided generative model to propose adjustments to the defending team's player positions and velocities—leaving the attacking team unchanged—such that the predicted shot probability is reduced. We chose to focus on the defending team to match a real-world workload: typically, a corner setup is dictated by the attacking team, after which the defensive team needs to respond to this setup in an optimal way. For each of these corners, we showed the raters the two versions (original and TacticAI-adjusted) side-by-side. We stated that the corner on the left-hand side is a reference corner, asking the raters to judge which of the defending team's players are in a better or worse position, considering the adjustments on the right-hand side. We also asked the raters to judge whether the right-hand side corner is, overall, a better or worse situation than the left-hand side for the defensive team. To control for bias, we randomly select a subset of corner setups for which we reverse the two versions, making the TacticAI suggestion the reference corner. The raters focused on a variety of salient features in the suggestions, including but not limited to: whether defenders are better at tracking an attacker's run, whether it is better for them to move or stand still, and the positioning of the goalkeeper. There have been two situations where an adjustment was judged as "possibly useful", depending on the adjusted player's profile. For example, certain runs were only deemed likely to succeed if performed by defenders of high physical capability or fitness. As the TacticAI variant we trained here did not have access to player features beyond height and weight, such nuances were out of scope for this study, and we did not consider them to be salient observations. Overall, as outlined in the Results section, the raters were overwhelmingly in favour of TacticAI's suggestions, demonstrating a high degree of inter-rater agreement. We illustrate four of the most salient situations where TacticAI's suggestions were deemed significant in Figure 5; in one of these situations, **ten** defensive players were considered usefully adjusted, which is nearly the entire defending team! Further, this kind of position-adjustment system has been deemed a highly useful tool for an analyst or coach to leverage by all of our raters. Even the opportunity to view such suggestions side-by-side with a reference corner was considered to be useful, as it prompted the rater to immediately consider strategic variations. Since the guided generation system was deliberately designed to offer subtle modifications to player coordinates and velocities, the raters deemed such a system most useful to help detect players that have likely not been following the instructions of their coaches, and neglected the tactical play. This can then lead to targeted interventions by the coaches—either to exploit a weakness posed by an opposing player that tends to ignore instructions, or to improve coaching of the team's player if they are found to be neglecting the determined tactics. Coupled

with the positive outcome of Task 1, and the finding that the suggested adjustments are generally hard to distinguish from real corner situations, we conclude Task 4's question to be answered in the affirmative.

## Additional Ablation and Stability Studies

In this section, we provide several further ablation studies on TacticAI's dataset, input representation, architectural choices and output stability, to further supplement our results and justify some of our design choices.

Firstly, as discussed in the main paper, our training and test datasets are randomly sampled across all corner kick situations. While we find this to be a fair way to evaluate the capabilities of our system in capturing tactical subtlety, we also acknowledge that tactics may be subject to drifting over time, and that there may be multiple similar tactical executions in the same game. To evaluate how TacticAI systems would perform under temporal drift, we re-ran all of our ablation studies under an 80%–20% *temporal split*, where the 20% of the corner kicks from games that occurred latest in our dataset would be used for testing. Our results on the temporal split are summarised in Supplementary Table 2 (for receiver prediction) and Supplementary Table 3 (for shot prediction).

Our findings broadly indicate that utilising a temporal split does not affect the relative ordering of our baselines—hence, our conclusions about TacticAI design still hold in this regime. Further, while all baselines experience slight regressions in the receiver prediction performance, the $D_2$-symmetric model is the most stable; its top-3 accuracy is reduced by $< 3\%$, whereas the top-3 accuracies of non-geometric GNNs are reduced by $\geq 5\%$. We suspect that this stability is a direct consequence of the $D_2$-equivariant model's enforcement of identical outputs for symmetric situations, which might make the model less sensitive to repetitions of similar strategies in the training data.

Another way in which the stability of the TacticAI system is worth probing is within its guided generation model. While the ratings we received by experts already indicate that these generations are hard to distinguish from real corner kick situations, we quantitatively evaluate to what extent the generative model may produce unrealistic player positions or velocities. These results are summarised in Supplementary Figure 6.

Our results indicate that TacticAI mainly recommends subtle player position changes of $< 1$m, being more conservative for defending players, as they tend to stay confined within the box or near it. Further, over a sample of $1,100$ player adjustments, we found that there were no tactical recommendations which would move the player out of the bounds of the pitch[1].

Similarly, we find that TacticAI recommends subtle changes to player velocity, with slightly increased variance towards larger speeds—which corresponds to our raters' remarks that TacticAI is a potent system for detecting players who may be neglecting their strategic roles. Further, we find that TacticAI only recommends speeds larger than ones seen in the training data for 0.3% of the players—generally suggesting that its recommended velocities are within plausible ranges.

Lastly, to further probe the capabilities of our choices in designing TacticAI's base model, we provide additional ablations in Supplementary Table 4 (for receiver prediction) and Supplementary Table 5 (for shot prediction).

Aside from the already-mentioned height/weight ablation, we also compared our models against heterogeneous GATs [22], and a Transformer-based encoder [23, 24]. The first comparison is de-

[1]Provided they were not already out-of-bounds in the real corner—which happened in only three cases overall.

signed to check whether the heterogeneous graph structure of the football teams can be exploited in different ways, whereas the other seeks to validate whether the GATv2 attention is competitive against the more popular dot-product self-attention. In both cases, we either observe no significant differences, or a performance degradation in the ablated architectures, further confirming our choices in graph and architecture design for TacticAI.

## Supplementary Figures

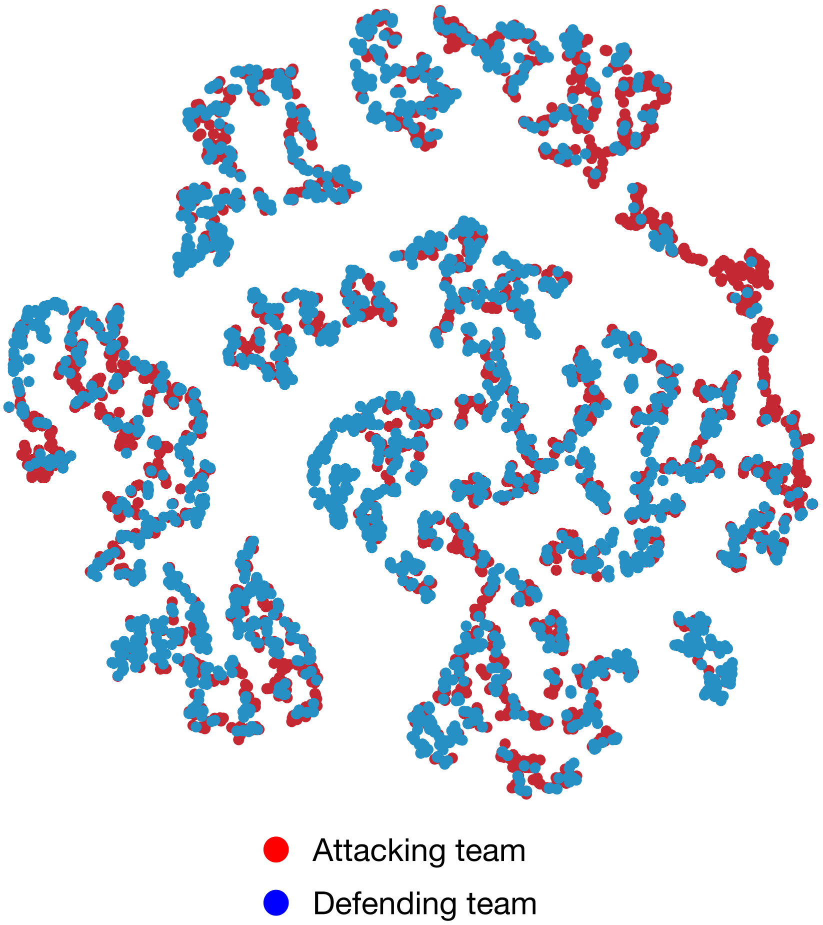


Supplementary Figure 1: ***t*-SNE embeddings of raw input features.** For the same set of corner kick samples used in the *t*-SNE visualisation in Figure 2, we visualise their raw input features with *t*-SNE. The *t*-SNE embeddings of the attacking and defending setups are not clearly separable.

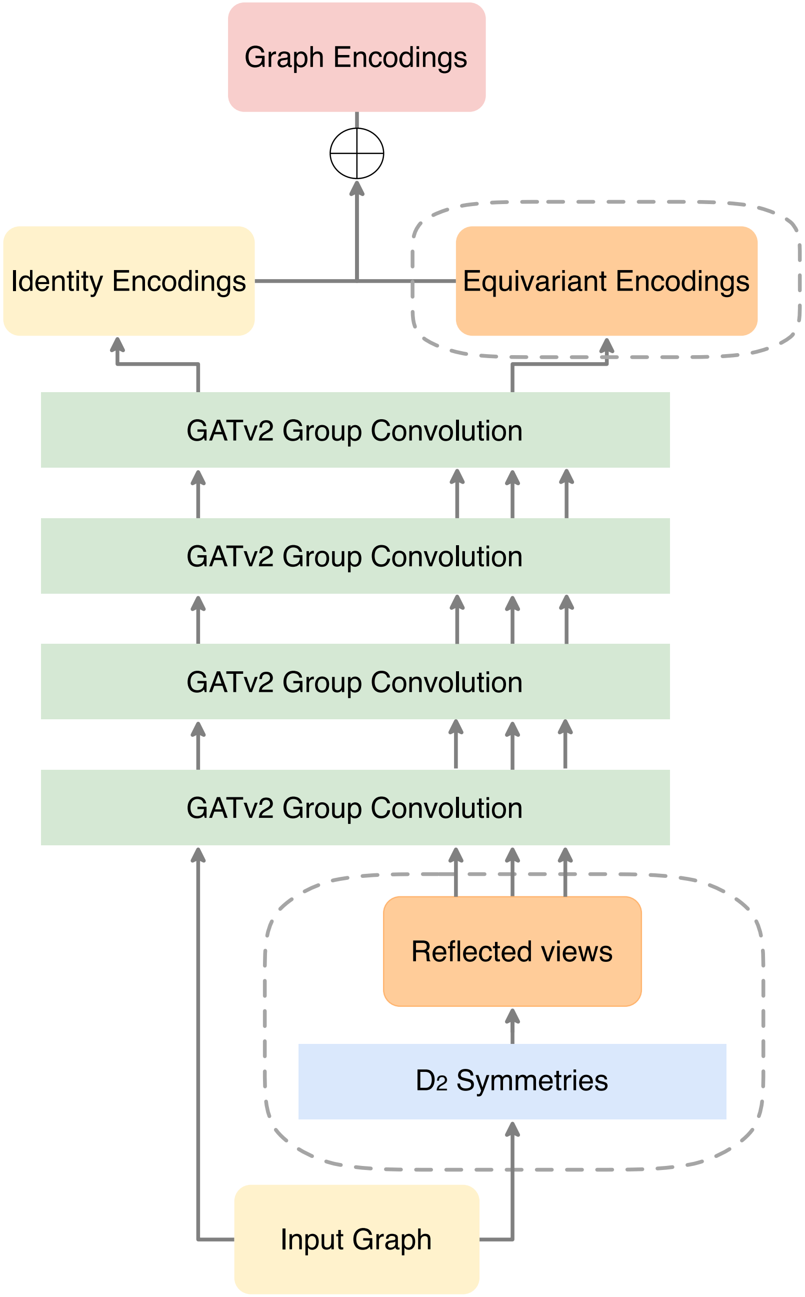


Supplementary Figure 2: **The encoder architecture of TacticAI's predictive and generative component models.** For each input graph, we first generate three $D_2$-reflected views. Secondly, we process them via a sequence of four GATv2 [25] group convolution layers. The actual number of layers may be different in different tasks (Supplementary Table 1). Finally, we aggregate the encodings of the input graph (identity encodings) and the encodings of its corresponding reflected views (equivariant encodings) through a weighted sum to obtain the final graph encodings. Specifically, for the receiver and shot prediction tasks (which are fully invariant), we take the mean of the encodings, and for the guided generation task (which is equivariant), we set the weight of the identity encodings to 1.0 and zero out the equivariant encodings. The graph encodings are further processed with respect to the corresponding tasks (see Network Architectures in the Methods section.)

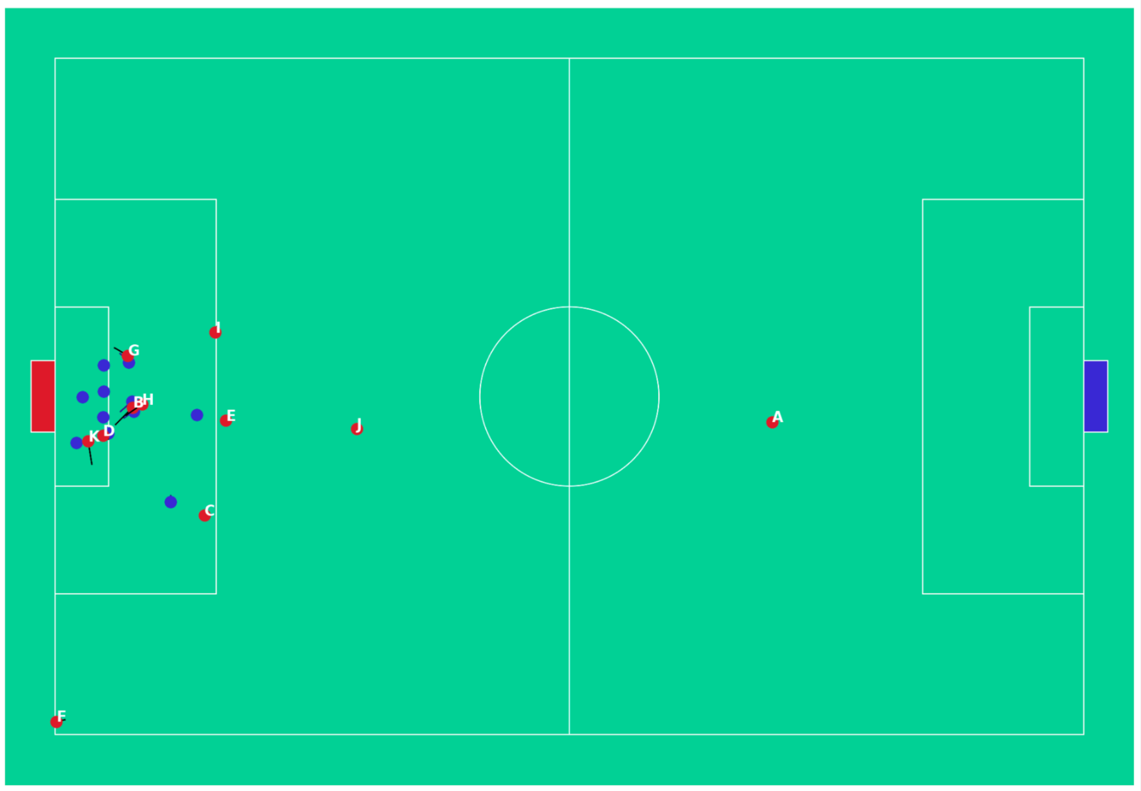


Supplementary Figure 3: **Sample of Task 1 and 2 in the case study.** Each corner kick sample in this task is presented in a 2D "bird's eye" view, in which blue and red dots represent defending and attacking players, respectively, and the length of the arrow attached to each dot indicates their velocity. During the task, for each sample, we first ask the human experts to identify whether the sample is a real corner kick or a generated one by TacticAI. Next, they are asked to identify the most likely receivers among the attacking team's players.

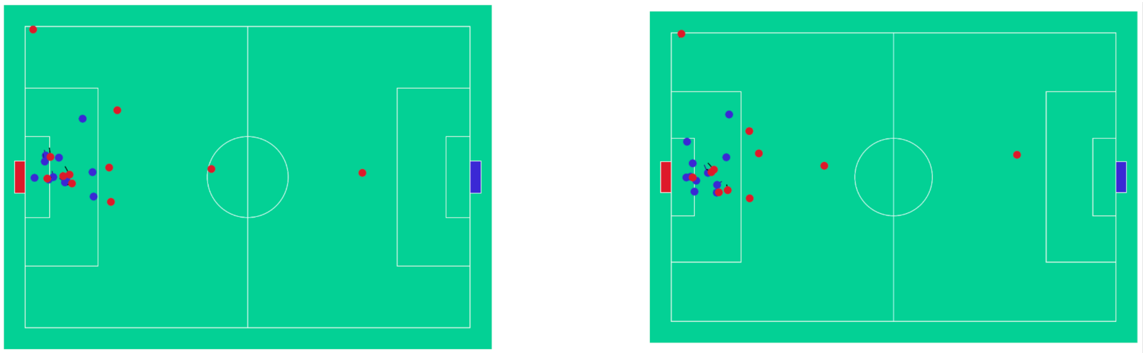

Supplementary Figure 4: **Sample of Task 3 in the case study.** In this task, we present a pair of corner kicks in each task sample, one of which represents the reference corner kick, and the other represents a retrieved corner, taken as the closest sample in distance, either in the TacticAI latent space or in the raw feature space. The human raters are asked to rate whether the pairs are similar with respect salient patterns.

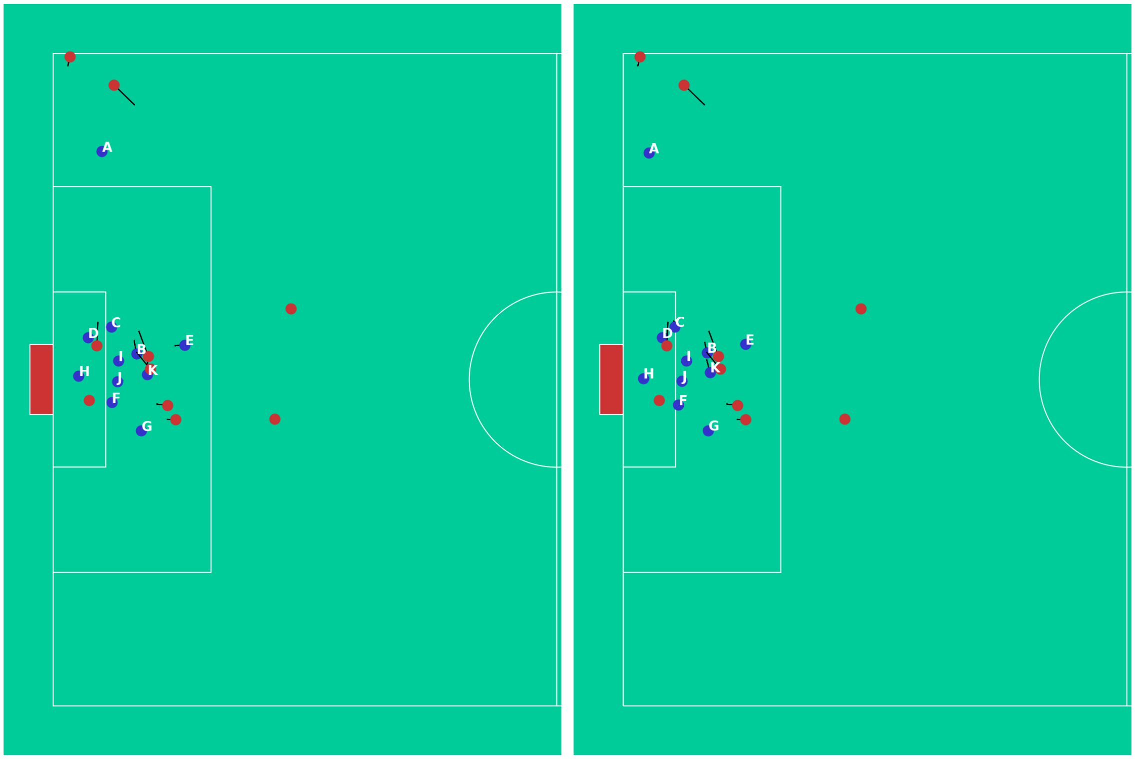


Supplementary Figure 5: **Sample of Task 4 in the case study.** In this task, the human raters are given pairs of corner kick samples, each of which consists of a reference corner kick sample and its adjustment generated by TacticAI. Then, the raters are asked to identify whether there are salient improvements in the adjustment and also to give specific account to those improvements as well. For the adjustment in each pair, we rate it as +1 if the rater confirms that overall adjustments are constructive, 0 if no distinct difference, or −1 if destructive.

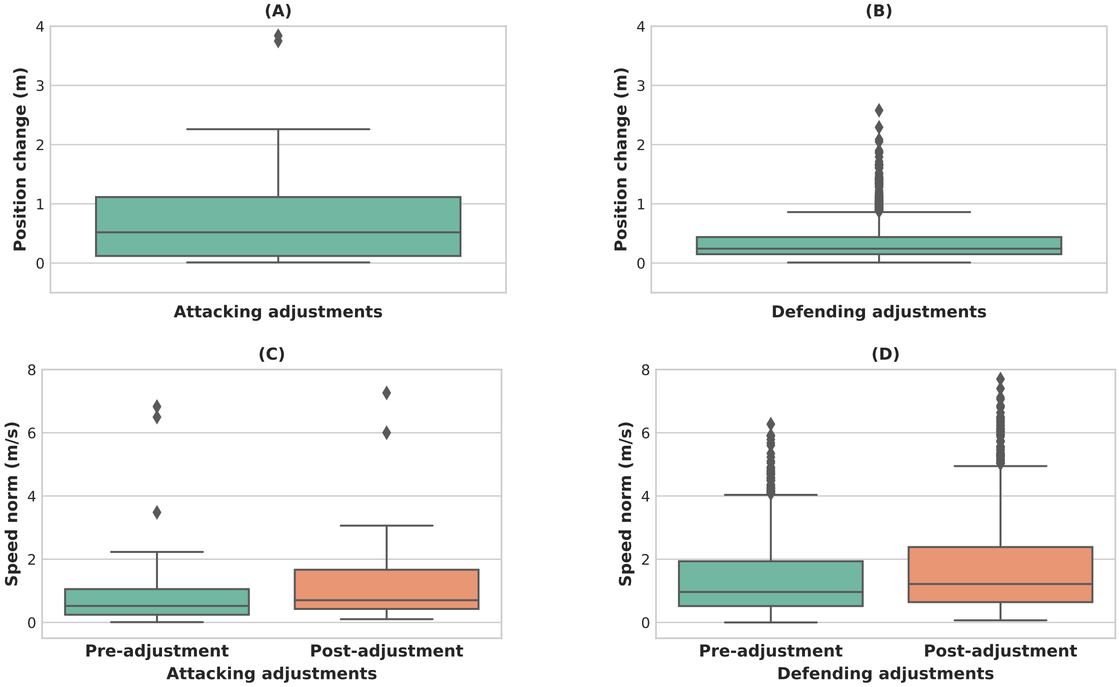


Supplementary Figure 6: **Stability analysis for tactic refinement**. In addition to evaluating the realism of the tactic adjustments recommended by TacticAI in the case study, we also quantitatively analyse the adjustments with two metrics: 1) the change of player positions in meters (m), and 2) the change of player speed norms in meters per second (m/s). In particular, we plot these quantities over 100 TacticAI-generated attacking and defending team adjustments. First, we see that the suggested position changes are within feasible ranges (up to 4m), for both the attacking (**A**) or defending (**B**) teams. Secondly, we observe that the position variation of the attacking teams (**A**) is larger than that of the defending teams (**B**), which is likely due to the relatively restricted range of positions for defending players in a corner kick. Finally, when it comes to velocity changes, we see that, in general, TacticAI suggests faster movements for both attacking (**C**) and defending (**D**) teams. These changes are still feasible in practice, as suggested by the feedback received from the expert human raters.

## Supplementary Tables

| Hyperparameter | Receiver Prediction | Shot Prediction | Guided Generation |
|---|---|---|---|
| Batch size | 256 | 128 | 128 |
| Learning rate | 1e-4 | 1e-4 | 5e-5 |
| $L^2$ weight decay | 1e-4 | 0. | 1e-4 |
| Adam optimiser $\beta_1$ | 0.9 | 0.9 | 0.9 |
| Adam optimiser $\beta_2$ | 0.999 | 0.999 | 0.999 |
| Adam optimiser $\epsilon$ | 1e-8 | 1e-8 | 1e-8 |
| # Graph attention layers | 4 | 2 | 2 |

Supplementary Table 1: **Hyperparameters for training TacticAI's components.** We list the hyperparameters with which we obtain TacticAI's best performing component models. The models are selected according to their evaluation losses. In particular, for guided generation, attacking and defensive generations are trained as two models, and share a same set of hyperparameters.

| **Model** | **Average Top-3 Accuracy** | |
|---|---|---|
| | Random training-test split | Temporal training-test split |
| Random | 0.136 | 0.136 |
| CNN [15] | $0.364 \pm 0.031$ | $0.358 \pm 0.061$ |
| Deep Sets [26] | $0.713 \pm 0.022$ | $0.649 \pm 0.073$ |
| MPNN [27] | $0.723 \pm 0.017$ | $0.664 \pm 0.033$ |
| GATv2 [28, 25] | $0.748 \pm 0.021$ | $0.698 \pm 0.047$ |
| GATv2 + $D_2$ frame averaging | $0.780 \pm 0.011$ | $0.749 \pm 0.078$ |
| GATv2 + $D_2$ group convolution [29] | $\mathbf{0.782} \pm 0.039$ | $\mathbf{0.753} \pm 0.050$ |

Supplementary Table 2: **Ablation results for receiver prediction.** We use top-3 accuracy as the metric, and we ablate our methods with two 80%–20% training-test split schemes: a *randomised* sampling of corner kicks, and a *temporal* split, where models are trained with older corner kicks and are evaluated on the 20% latest ones. In particular, the random split assumes that the corner kick samples are independently and identically distributed (i.i.d.), and the temporal split alleviates temporal correlations between training and test samples because the more adjacent the tactics one team use, the more similar they would be. On both splits, we observe that a graph representation with Deep Sets [26] outperforms a convolutional neural network (CNN [15]), which does not leverage a graph representation. Secondly, instead of modelling each player node in isolation without considering adjacency information with Deep Sets, we augment each pair of player nodes with an edge to indicate whether they are on the same team, and process the resulting fully connected graphs with various GNNs (MPNN [27] and GATv2 [25]). This yields another performance gain in Top-3 prediction accuracy, particularly using GATv2. Finally, equipping the GATv2 with a $D_2$ group convolution yields our best performing model for receiver prediction.

| **Model** | **Average $F_1$ Score** | |
|---|---|---|
| | Random training-test split | Temporal training-test split |
| Random | 0.500 | 0.500 |
| GATv2 [28, 25] (unconditional) | $0.521 \pm 0.027$ | $0.504 \pm 0.010$ |
| GATv2 + receiver conditional | $0.677 \pm 0.036$ | $0.659 \pm 0.074$ |
| GATv2 + receiver conditional + $D_2$ group convolution [29] | $\mathbf{0.712} \pm 0.011$ | $\mathbf{0.716} \pm 0.009$ |

Supplementary Table 3: **Ablation results for shot prediction.** We use the $F_1$ score as the metric, because the dataset used to develop the shot prediction component model is imbalanced with a positive-to-negative ratio of 0.21. Instead of directly predicting the unconditional probability of a successful shot attempt, we predict the shot probability conditioned on the receiver of the corner kick, which yields a clear improvement in both random and temporal splits.

| Model | Average Top-3 Accuracy |
|---|---|
| GATv2 [28, 25] + $D_2$ group convolution [29] | $0.782 \pm 0.039$ |
| Without height and weight features | $0.763 \pm 0.060$ |
| With Heterogeneous GAT [22] encoder | $0.785 \pm 0.043$ |
| With Transformer attention [24] | $0.779 \pm 0.051$ |

Supplementary Table 4: **Architecture and feature ablation results for receiver prediction.** All models presented in this ablation apply $D_2$ symmetries and group convolution [29]. We ablate the usage of player heights and weights, the usage of heterogeneous graph encoders, and dot-product attention from Transformers.

| Model | Average $F_1$ Score |
|---|---|
| GATv2 + $D_2$ group convolution | $0.712 \pm 0.011$ |
| Without height and weight features | $0.676 \pm 0.063$ |
| With Heterogeneous GAT [22] encoder | $0.668 \pm 0.067$ |
| With Transformer attention [24] | $0.715 \pm 0.010$ |

Supplementary Table 5: **Architecture and feature ablation results for shot prediction.** All models presented in this ablation apply $D_2$ symmetries and group convolution [29]. We ablate the usage of player heights and weights, the usage of heterogeneous graph encoders, and dot-product attention from Transformers.

# Supplementary References


[1] Adrien Deliege, Anthony Cioppa, Silvio Giancola, Meisam J Seikavandi, Jacob V Dueholm, Kamal Nasrollahi, Bernard Ghanem, Thomas B Moeslund, and Marc Van Droogenbroeck. Soccernet-v2: A dataset and benchmarks for holistic understanding of broadcast soccer videos. In *Proceedings of the IEEE/CVF conference on computer vision and pattern recognition*, pages 4508–4519, 2021.

[2] Javier Fernández, Luke Bornn, and Dan Cervone. Decomposing the immeasurable sport: A deep learning expected possession value framework for soccer. In *13th MIT Sloan Sports Analytics Conference*, 2019.

[3] FR Goes, LA Meerhoff, MJO Bueno, DM Rodrigues, FA Moura, MS Brink, MT Elferink-Gemser, AJ Knobbe, SA Cunha, RS Torres, et al. Unlocking the potential of big data to support tactical performance analysis in professional soccer: A systematic review. *European Journal of Sport Science*, 21(4):481–496, 2021.

[4] Benedict Low, Diogo Coutinho, Bruno Gonçalves, Robert Rein, Daniel Memmert, and Jaime Sampaio. A systematic review of collective tactical behaviours in football using positional data. *Sports Medicine*, 50:343–385, 2020.

[5] Luca Pappalardo, Paolo Cintia, Alessio Rossi, Emanuele Massucco, Paolo Ferragina, Dino Pedreschi, and Fosca Giannotti. A public data set of spatio-temporal match events in soccer competitions. *Scientific data*, 6(1):236, 2019.

[6] Robert Rein and Daniel Memmert. Big data and tactical analysis in elite soccer: future challenges and opportunities for sports science. *SpringerPlus*, 5(1):1–13, 2016.

[7] Steffen Lang, Raphael Wild, Alexander Isenko, and Daniel Link. Predicting the in-game status in soccer with machine learning using spatiotemporal player tracking data. *Scientific Reports*, 12(1):16291, 2022.

[8] Jacob Newman, Andrew Sumsion, Shad Torrie, and Dah-Jye Lee. Automated Pre-Play Analysis of American Football Formations Using Deep Learning. *Electronics*, 12(3):726, 2023.

[9] Hyeonah Cho, Hyunyoung Ryu, and Minseok Song. Pass2vec: Analyzing soccer players' passing style using deep learning. *International Journal of Sports Science & Coaching*, 17(2):355–365, 2022.

[10] Yutaro Honda, Rei Kawakami, Ryota Yoshihashi, Kenta Kato, and Takeshi Naemura. Pass Receiver Prediction in Soccer using Video and Players' Trajectories. In *2022 IEEE/CVF Conference on Computer Vision and Pattern Recognition Workshops (CVPRW)*, pages 3502–3511, 2022.

[11] Ryota Goka, Yuya Moroto, Keisuke Maeda, Takahiro Ogawa, and Miki Haseyama. Prediction of Shooting Events in Soccer Videos Using Complete Bipartite Graphs and Players' Spatial-Temporal Relations. *Sensors*, 23(9):4506, 2023.

[12] Leander Forcher, Tobias Beckmann, Oliver Wohak, Christian Romeike, Ferdinand Graf, and Stefan Altmann. Prediction of defensive success in elite soccer using machine learning-Tactical analysis of defensive play using tracking data and explainable AI. *Science and Medicine in Football*, pages 1–16, 2023.

[13] Dominik Raabe, Reinhard Nabben, and Daniel Memmert. Graph representations for the analysis of multi-agent spatiotemporal sports data. *Applied Intelligence*, 53(4):3783–3803, 2023.

[14] Michael Stöckl, Thomas Seidl, Daniel Marley, and Paul Power. Making offensive play predictable-using a graph convolutional network to understand defensive performance in soccer. In *Proceedings of the 15th MIT sloan sports analytics conference*, volume 2022, 2021.

[15] Javier Fernández and Luke Bornn. Soccermap: A deep learning architecture for visually-interpretable analysis in soccer. In *Machine Learning and Knowledge Discovery in Databases. Applied Data Science and Demo Track: European Conference, ECML PKDD 2020, Ghent, Belgium, September 14–18, 2020, Proceedings, Part V*, pages 491–506. Springer, 2021.

[16] Ondřej Hubáček, Gustav Sourek, and Filip Železný. Deep Learning from Spatial Relations for Soccer Pass Prediction. In *MLSA@PKDD/ECML*, 2018.

[17] Longyuan Li, Jian Yao, Li Wenliang, Tong He, Tianjun Xiao, Junchi Yan, David Wipf, and Zheng Zhang. GRIN: Generative Relation and Intention Network for Multi-agent Trajectory Prediction. In M. Ranzato, A. Beygelzimer, Y. Dauphin, P.S. Liang, and J. Wortman Vaughan, editors, *Advances in Neural Information Processing Systems*, volume 34, pages 27107–27118. Curran Associates, Inc., 2021.

[18] Anthony Sicilia, Konstantinos Pelechrinis, and Kirk Goldsberry. Deephoops: Evaluating micro-actions in basketball using deep feature representations of spatio-temporal data. In *Proceedings of the 25th ACM SIGKDD International Conference on Knowledge Discovery & Data Mining*, pages 2096–2104, 2019.

[19] Rui Luo and Vikram Krishnamurthy. Who you play affects how you play: Predicting sports performance using graph attention networks with temporal convolution. *arXiv preprint arXiv:2303.16741*, 2023.

[20] Manuel Bassek, Dominik Raabe, Alexander Banning, Daniel Memmert, and Robert Rein. Analysis of contextualized intensity in men's elite handball using graph-based deep learning. *Journal of Sports Sciences*, 41(13):1299–1308, 2023.

[21] Cleyton R Silva, Michael Bowling, and Levi HS Lelis. Teaching people by justifying tree search decisions: An empirical study in curling. *Journal of Artificial Intelligence Research*, 72:1083–1102, 2021.

[22] Xiao Wang, Houye Ji, Chuan Shi, Bai Wang, Yanfang Ye, Peng Cui, and Philip S Yu. Heterogeneous graph attention network. WWW '19, page 2022–2032, New York, NY, USA, 2019. Association for Computing Machinery.

[23] Ashish Vaswani, Noam Shazeer, Niki Parmar, Jakob Uszkoreit, Llion Jones, Aidan N Gomez, Łukasz Kaiser, and Illia Polosukhin. Attention is all you need. *Advances in neural information processing systems*, 30, 2017.

[24] Luis Müller, Mikhail Galkin, Christopher Morris, and Ladislav Rampášek. Attending to graph transformers. *arXiv preprint arXiv:2302.04181*, 2023.

[25] Shaked Brody, Uri Alon, and Eran Yahav. How attentive are graph attention networks? In *International Conference on Learning Representations*, 2022.

[26] Manzil Zaheer, Satwik Kottur, Siamak Ravanbakhsh, Barnabas Poczos, Russ R Salakhutdinov, and Alexander J Smola. Deep Sets. In I. Guyon, U. Von Luxburg, S. Bengio, H. Wallach, R. Fergus, S. Vishwanathan, and R. Garnett, editors, *Advances in Neural Information Processing Systems*, volume 30. Curran Associates, Inc., 2017.

[27] Justin Gilmer, Samuel S. Schoenholz, Patrick F. Riley, Oriol Vinyals, and George E. Dahl. Neural Message Passing for Quantum Chemistry. In Doina Precup and Yee Whye Teh, editors, *Proceedings of the 34th International Conference on Machine Learning*, volume 70 of *Proceedings of Machine Learning Research*, pages 1263–1272. PMLR, 06–11 Aug 2017.

[28] Petar Veličković, Guillem Cucurull, Arantxa Casanova, Adriana Romero, Pietro Liò, and Yoshua Bengio. Graph attention networks. In *International Conference on Learning Representations*, 2018.

[29] Taco Cohen and Max Welling. Group equivariant convolutional networks. In *International conference on machine learning*, pages 2990–2999. PMLR, 2016.
